# Hourly Water Level Forecasting in an Hydroelectric Basin Using Spatial Interpolation and Artificial Intelligence

**DOI:** 10.3390/s23010203

**Published:** 2022-12-25

**Authors:** Mauro Tucci

**Affiliations:** Department of Energy, Systems, Territory and Construction Engineering, University of Pisa, Largo Lucio Lazzarino 1, 56122 Pisa, Italy; mauro.tucci@unipi.it

**Keywords:** hydroelectric basin modelling, spatial interpolation, neural networks, Kriging

## Abstract

In this work, a new hydroelectric basin modelling approach is described and applied to the Pontecosi basin, Italy. Several types of data sources were used to learn the model: a number of weather stations, satellite observations, the reanalysis dataset, and basin data. With the goal of predicting the water level of the basin, the model was composed by three cascade modules. Firstly, different spatial interpolation methods, such as Kriging, Radial Basis Function, and Natural Neighbours, were compared and applied to interpolate the weather stations data nearby the basin area to infer the main environmental variables (air temperature, air humidity, precipitation, and wind speed) in the basin area. Then, using these variables as inputs, a neural network was trained to predict the mean soil moisture concentration over the area, also to improve the low availability due to satellite orbits. Finally, a non-linear auto regressive exogenous input (NARX) model was trained to simulate the basin level with different prediction horizons, using the data from the previous modules and past basin data (water level, discharge flow rate, and turbine flow rate). Accurate predictions of the basin water level were achieved within 1 to 6 h ahead, with mean absolute errors (MAE) between 2 cm and 10 cm, respectively.

## 1. Introduction

Water is one of most important resources of the world. Monitoring rivers, basins, and seas is a very important challenge for many different applications, from agricultural utilization to electric energy production [1]. Wrong management can lead to natural calamities, such as floods and dry rivers [2,3]. From an energy production point of view, basin level, and turbine water flow are deeply linked, and weather conditions can influence not only the basin level but also the plant operations [4]. Traditional physical models often struggle with parameters evaluation, and in any case a large measurement campaign must be conducted in the geographic area of study. Machine learning and black box modelling can overcome this problem, and for this reason a large number of studies in the literature related to water level forecasting and monitoring focus on artificial intelligence methods [5,6]. Main findings in [6] are:

- The results indicated that the applied forecasting models are efficient and trustworthy,
- Cross-station modeling revealed an optimistic modeling strategy for learning transfer modeling of using information of nearby site.

The second point above is one of the main motivations of this work.

A monthly [7,8,9,10,11] or daily [12,13,14,15] average water level time series is usually considered for lakes and basins, while a prediction within hours [16,17] or even minutes [18] is often necessary in the case floods. To predict water levels, most of these works use past measurements, although from different sources, of the water level itself, in an auto-regressive fashion. Some works also consider rainfall [14,18], and temperature as well [8]. Other than using local sensors, water level can be monitored by satellite altimeters [15] or radars [10,15]. Some of the cited works use feed-forward neural networks to predict the water level [9,16]. Very good prediction performance is reported in [16], with errors between 0.06 m and 0.12 m for 1 to 2 hours-ahead, respectively, using water level data from multiple stations, indicating that adding more data sources can be beneficial. Other works use support vector machines (SVM) [7,12] or adaptive neuro-fuzzy inference system (ANFIS) [8,13]. In the case of hourly (or less) forecast horizons, NARX neural networks are better suited [17,18]. Some works [11] focus on clustering algorithms and monitoring [10], while recently developed deep learning methods have also been applied [15,19]. In particular in [19] the LSTM (long short-term memory) approach, which is recent methodology belonging to the deep learning paradigm, was found to be more effective than SVM. Other methods, such as boosted decision trees and Bayesian linear regression were reported to be successful for daily water level prediction in a hydroelectric basin using also rainfall data as an input [14]. Successful applications of machine learning approaches for time series forecasting in the energy sector can be found also in the case of electricity price prediction [20], where Kalman filter and Echo State Networks are used, electrical load prediction [21], where a variant of the K-Nearest Neighbours algorithm is proposed, as well as prediction of the power generation from photovoltaic plants [22], and wind plants [23]. In [24], the Kalman filter is successfully applied as a data assimilation scheme for water level forecasting.

In this work, we focus on the Garfagnana valley hydroelectrical system, which is composed by several basins and power plants connected to each other. Managing the water resource all over the valley is a complex task, and knowing in advance the level of the basins would be of great convenience. Moreover, the need of an hydrological model is justified by the environmental challenges that nowadays are constantly on the spotlight: knowing the status of the water resource can be crucial to avoid natural calamities and to maintain the environmental flow.

The main objective of this work is to develop an accurate hourly forecasting model of the water level in a hydroelectric basin, exploiting a much larger number of data sources with respect to most of the literature.

In fact, a first contribution of this work is that a considerably large number of variables was used to create the model with respect to many other works related to water level forecasting. The data include several weather variables (temperature, humidity, precipitation, and wind speed) from 14 weather stations (WS) scattered throughout the geographical area of the basin (northwestern area of Tuscany), satellite measurements of the soil moisture concentration (SMC), reanalysis data of net solar radiance and snow depth, and hourly values of basin level, turbine flow rate and discharge flow rate. Moreover, data were collected for almost 19 consecutive months, 631 days, starting 6 June 2017, and ending 27 February 2019. The datasets and the variables were selected to address the main objective which is to improve the accuracy of the water level forecasting in the basin. Each one of the selected variables has a direct or indirect effect on the water level in the basin, where soil moisture concentration, precipitation, temperature, and air humidity are the most effective, besides the direct basin activity, such as turbine flow rate and discharge flow rate. Using a number of different sources was a main guideline of this project, intending to utilize all of the available data that could influence the outcome of the prediction, even if the expected influence was small.

A second contribution of this work is related to the articulate data preprocessing. In fact, some of the weather stations are several kilometers far from the basin. For this reason, the weather stations variables were spatially interpolated, considering the distance between each WS and the basin, to obtain an accurate average value in the basin area. In particular, three different spatial interpolation algorithms were tested (Kriging, Radial Basis Function, Natural Neighbour) and compared. Another problem is related to the low availability of the satellite SMC maps, which are obtained twice a week. To improve SMC availability, a neural network surrogate model was trained to predict SMC, using the spatially interpolated weather variables as inputs and the satellite SMC values as output.

As a final contribution, a state of the art NARX model was developed using all of the previously mentioned variables as inputs to predict the water level *h* hours ahead, for different values of the time horizon *h*. Hourly prediction of water levels, as well as NARX models, are most common in the case of flood prediction, but it can be certainly beneficial for the management of the hydroelectric basin for several operational reasons, especially in the case of several interconnected basins as in the case of the Garfagnana valley. As an example, an important activity in the hydroelectric basin is predictive maintenance [25,26], and an hourly prediction of the basin status can be conveniently used to perform condition monitoring and fault detection. In the previous work [25], the author proposed a self-organizing maps (SOM)-based methodology to condition monitoring and predictive maintenance in hydroelectric plants. In that work, a number of measured variables from the generations units equipment, such as currents, temperatures of the transformers oil and windings, sensor data from the electrical machines, dissolved gases, and many other variables, were used to learn the SOM model of the plant operation in nominal conditions. The model proposed in this work will be used as an additional module to aid the predictive maintenance algorithm to monitor the effective operation of the system and predict faults. In fact, having an accurate simulation of the water level in nominal conditions, allows to compare the real water level against the predicted water level, and a significant disagreement indicates an anomalous behaviour of the system.

In Section 2 the main characteristics of the hydrological system are presented, the input data of is presented and described in detail, the various models are formulated and their characteristics are highlighted. In Section 3, the results of the methods are presented and discussed. Section 4 reports the conclusions and future developments.

## 2. Materials and Methods

In this section, we introduce the main aspects of the Garfagnana hydrological system, whose dimension and complexity motivate the development of the accurate model presented in this work. After that, we present the main characteristics of the collected datasets, which consist in reanalysis data, weather stations data, satellite data, and basin data. Finally, we introduce the methodology, which is composed by three main modules: spatial interpolation of weather stations data, neural network modelling of satellite data, and basin modelling using NARX approach.

### 2.1. Garfagnana Hydrological System

**Figure 1:**
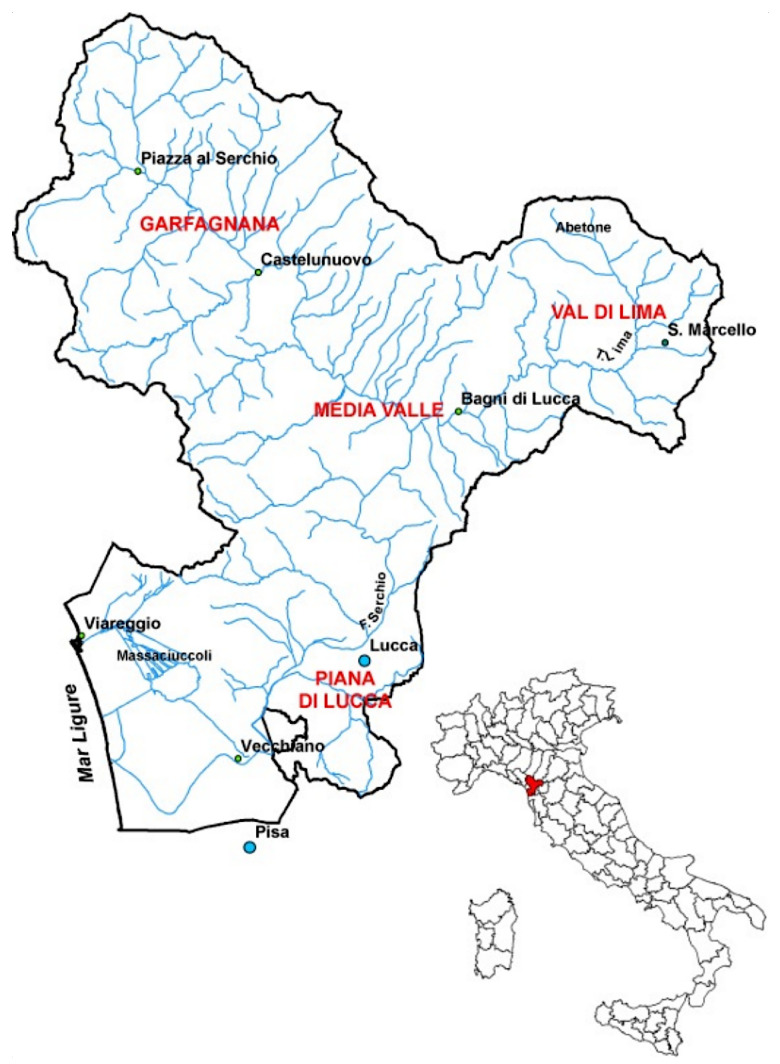
Overview of Serchio basin.

The Garfagnana hydrological system is the most important on the Tuscany region, the main river is Serchio (111 km), whose source is located in Monte Sillano (1864 m), and its basin has a total catchment surface of 1500 Km${}^{2}$. An overview of the Serchio basin is shown in Figure 1.

**Figure 2:**
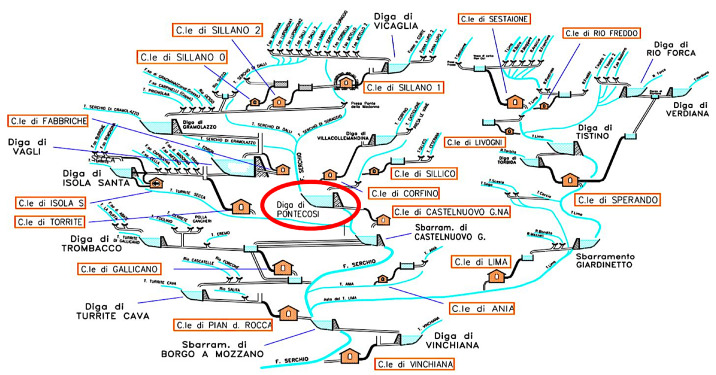
Hydro system in the area of Garfagnana.

Serchio goes through the Garfagnana valley until it reaches the Tirreno sea. The presence of large amount of hydro resource and its various distribution on the Garfagnana valley has influenced the realization of a complex system of river, basins, and power plants, as shown in Figure 2. The production of a specific power plant depends from the production of all the other power plants upstream. For example, if an upstream plant does not use the turbine for a certain period, the downstream basin will receive less water than usual. On the other side, the upstream basin will increase its water level. Optimizing the hydro resource all over the Garfagnana valley is one of the goals of this research. This kind of optimization is very complex because it requires a high level of coordination between all power plants of the area. The other main goal is to prevent the risk of flooding: the management of the hydro basin/plant must be handled in a safe way for the surrounding area. Knowing in advance the increase of water levels can help in taking the right decision and avoiding flooding. From a production point-of-view, having different basins at different altitudes requires a deep study for optimizing the global hydro resource. Being able to know the basin status in advance can strongly help the optimization process.

**Figure 3:**
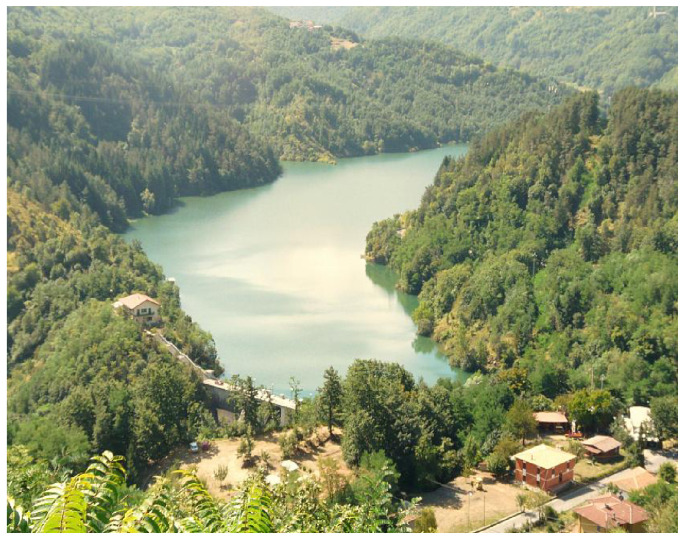
Pontecosi basin.

This work focuses on the Pontecosi basin which is located in the upper area of Garfagnana. An overview of the basin is shown in Figure 3. It is an artificial lake with a 30 m dam which serves as hydro-tank for the Castelnuovo hydroelectric power plant, located close to the lake. Castelnuovo power plant is the third biggest of the area; that justifies the importance of an accurate study of the hydro resource. In particular, Pontecosi basin is located at the heart (the middle-upper part) of the hydro-logical system, hence its good functioning is vital for the operation of the whole system, both upstream and downstream, for the reasons previously described. Having a good prediction of the water level of this basin is beneficial for the complete system as well.

From an environmental safety point of view the basin status is represented by the water level of the basin: upper and lower bounds are set by environmental laws. Going over the upper bound can cause floods; on the other side, a minimum level of water must be preserved to ensure the environmental flow (EF). The water level (neat head) in a hydroelectric basin depends on several variables: the flow used by the turbine, for example, is a human activity that influences the net head value. Higher is the turbine generation, higher is the amount of water withdrawn from the basin. Another aspect that influences the net head value is the EF, a minimum water outflow imposed by environmental laws to ensure a proper life quality of the river ecosystem. The EF must be guaranteed in all conditions by the owner of the plant.

It is clear that an accurate prediction of the water level, from a control/optimization point of view, is crucial to maintain the levels within the upper and lower bounds, to avoid flooding and the detriment of the environmental flow, respectively. In addition, an accurate prediction of the water level at a particular basin can be used to optimally plan in advance the operations in the upper and lower basins.

### 2.2. Datasets Characteristics

Data from several sources were used in this project, and of course data from such heterogeneous origins were affected by many quality issues, such as missing values, outliers, etc. Fortunately, most of the environmental data (90%) was of good quality, requiring a moderate preprocessing consisting mainly in the imputation of the missing values and the removal (and successive imputation) of the outliers.

#### 2.2.1. Reanalysis Dataset

Reanalysis dataset [27] were initially created to improve the performance of the Numerical Weather Prediction (NWP) models. NWP models need data about the initial state of weather variables: reanalysis data can provide it. Reanalysis datasets are generated by several data assimilation schemes and models. Data assimilation is a class of techniques which mixes different data sources, in order to obtain an output with less uncertainty than the original data. In this case, data assimilation uses machine learning (ML) algorithms instead of physical laws: this is mainly pushed by the complexity of the laws that rule all the weather variables.

Reanalysis dataset contains a large set of weather variables, extended on a large date–time range. The decision to use also this kind of data source is related to the possibility of having more inputs to the basin status model, hence increasing its performance.

The dataset used in this work is the ERA-Interim [27] which is released by European Centre for Medium-Range Weather Forecasts (ECMWF). This dataset is based on a 2006 Integrated Forecast System (IFS). The main properties are: 4-D variational analysis, 80 km of horizontal resolution, and 60 vertical levels (from the surface to 0.1 hPa). Similarly to satellite based observations, reanalysis dataset is provided in a gridded way: a matrix is associated with a specified position (latitude and longitude) and time. In comparison with satellite data, reanalysis has a lower resolution (80 km vs. 5 km): the accuracy of reanalysis data on a small area around Pontecosi basin is lower with respect to satellite data. Considering this, the variables selected from the reanalysis dataset were:

- Net solar radiance,
- Snow depth.

These variables are less influencing the basin status, with respect to rain and soil moisture concentration (SMC), so the lower accuracy of these variables can be accepted.

#### 2.2.2. Weather Stations Data

Weather stations (WS) are the most direct way to measure a weather variable. Usually, a WS is a complex system of sensors including not only the sensing elements, but also a data acquisition and transmission system. High-quality weather stations are composed by expensive products, so usually they are installed in key points of the area to be observed. In this project, we considered the following four weather variables acquired from weather stations:

- Mean daily temperature (°C),
- Mean daily air humidity (%),
- Total daily precipitation (mm),
- Mean daily wind speed (km/h).

**Figure 4:**
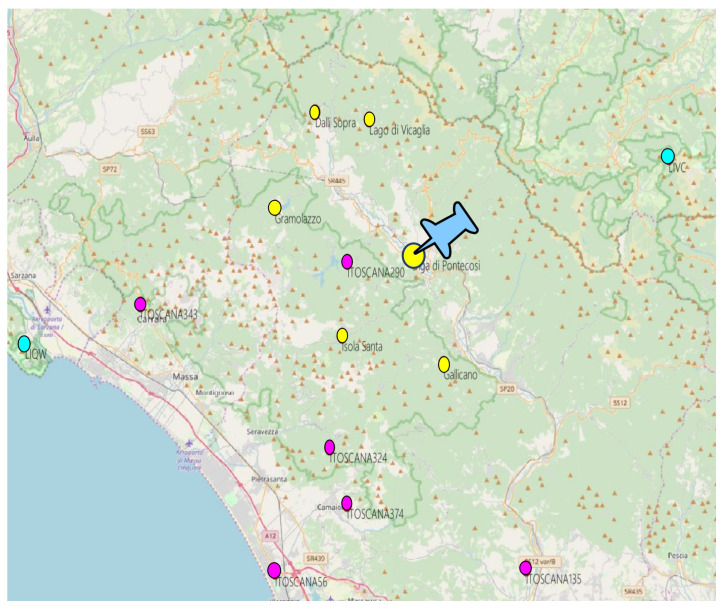
Weather stations positions. Yellow points are the plant owner stations, magenta points are Wunderground stations, and cyan points are Aeronautica Militare stations.

A total of 14 weather stations were used in this project, which are located in a large area nearby the Garfagnana Valley, as shown in Figure 4. The chosen weather stations belong to three different providers:

- Wunderground Personal Weather Stations (PWS) is a platform which offers weather observation datasets. PWS used in this work are located close to the Garfagnana area. The 6 weather stations are marked with a magenta circles in Figure 4, and they provide all 4 variables with worst-case availability of 90%. Wunderground dataset is the core of the input data of this project due to the high availability and proximity to the basin;
- Plant Owner Weather Stations. Plant owner made a weather measurements campaign in the Garfagnana valley. This dataset contains temperatures and air humidity from 6 weather stations located very close to Pontecosi area (yellow points), so they have high relevance with respect to the other providers;
- Aeronautica Militare (AM) Syrep stations are located far from the other stations, however additional two AM stations were selected to cover lack of availability of other providers (cyan points in the map). In addition, AM is the only authority which can certificate weather observations in Italy, so the data quality is generally very high.

#### 2.2.3. Satellite Data

**Figure 5:**
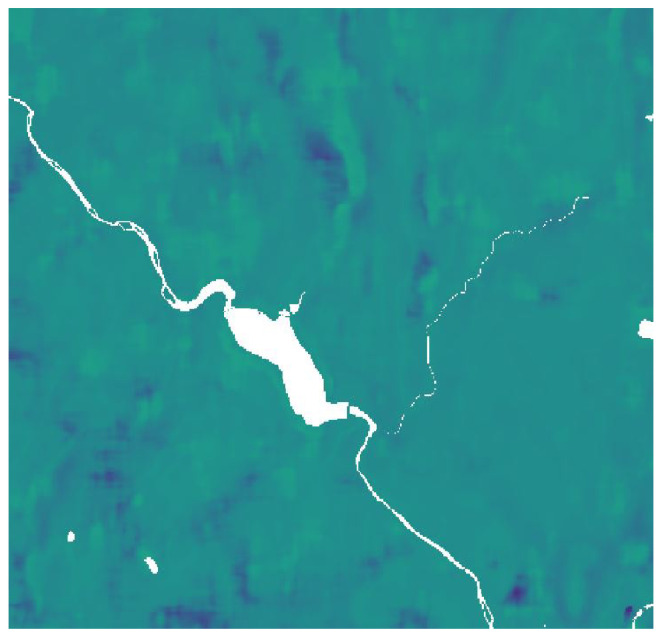
Soil moisture concentration satellite map around Pontecosi.

Nowadays, satellite observations are widely used in many fields, from the environmental study to military use. Satellite images are gridded data: it means that values are stored in a matrix, which is geo-referenced through a raster object. For longitudes between 80° North to 80° South, the raster object is usually based on a coordinate system called Universal Transverse Mercator (UTM), while for polar zones, Universal Polar Stereographic (UPS) projection is used. UTM system divides the Earth into 1200 zones: each of them has a size of 6° in longitude and 8° in latitude. The Pontecosi zone is named 32N. Inside the zone, each point is defined within a grid. The images used in this project have a 8074 × 9885 resolution. To select a smaller area than the original, latitude and longitude can be converted in UTM coordinates: so a smaller matrix can be extracted. In this project, satellite data images consist of soil moisture concentration (SMC) maps of the area. More in-depth, SMC values are mapped on a wide area of Tuscany, and a small area around Pontecosi can be extracted as shown in Figure 5. Satellite SMC images are available only twice a week. Averaging the values in the image a single scalar SMC value can be obtained, that represents the average soil moisture concentration around the basin.

#### 2.2.4. Basin Data

Pontecosi basin data were necessary to generate a basin model. Variables included in this dataset are:

- Basin level,
- Turbine flow rate,
- Discharge flow rate.

Each variable is given with a hourly time-step. It is important to note that these data contain several discharge flow events, which are major events during which the discharge flow outlet is activated to prevent flooding. The amount of water released by the basin during this kind of event is huge and it deeply influences the basin level.

### 2.3. Spatial Interpolation of Weather Stations Data

Point-wise data provided by Weather Stations (WS) cannot efficiently describe a weather variable in a large area. On the other hand, simply calculating an average value of the variables measured by several WS (which are several kilometers apart) can lead to large estimation errors. In these cases, the recommended practice is to use a spatial interpolator [28], which is a mathematical tool that can express a relationship between the spatial observations of a variable to predict its value in a different location. There is a large variety of spatial interpolators. In particular, Pontecosi basin is somewhat in a barycentric position with respect to all of the selected weather stations, then the nearest stations are expected to have a larger influence, while stations further away may have less influence. In order to accurately evaluate the relative weight of each weather station, a spatial interpolation algorithm is needed.

In this work we considered three main algorithms:

- Kriging [29];
- Radial Basis Function [30];
- Natural Neighbour [31].

The motivation of the choice of these three algorithms is that they represent three different main classes of spatial interpolation algorithms and they are extensively applied in the literature, being effective in a large number of applications. Kriging and Natural Neighbour make the prediction as a weighted linear combination of the values measured at the weather stations, while Radial Basis Function interpolates using Gaussian functions placed at the weather stations location.

**Figure 6:**
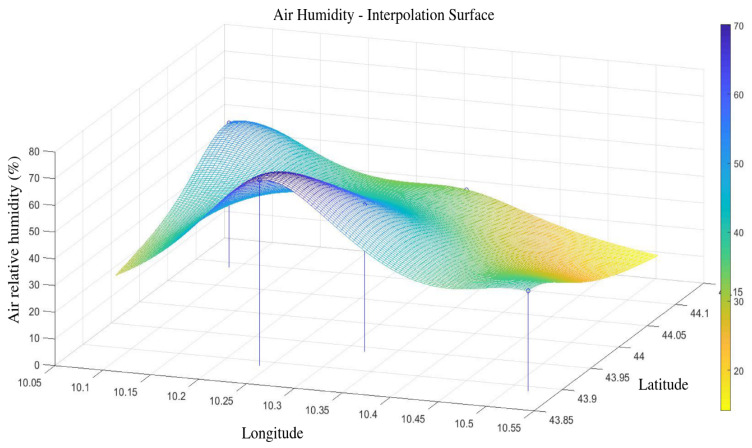
Kriging interpolation of air humidity over the area of interest in an r day. Vertical lines localize the weather stations.

The interpolated variable can be predicted in a specific new location as shown in Figure 6, most precisely, the interpolated values can be averaged over a specific area around the basin.

#### 2.3.1. Kriging Interpolation

The term “Kriging” comes from Danie Krige, an engineer who first developed this particular method. Several variants of the the Kriging algorithm exist: in this work Ordinary Kriging is used. In the following, the *n* measurement sites are defined as “sample points”.

The Kriging prediction $\hat{z}\left(x_{0}\right)$ of a scalar quantity $z(x_{0})$ at an unobserved location $x_{0}$ is a linear combination of the observed values $z(x_{i})$ at sample points $x_{i}$ with scalar weights $w_{i}$, i=1⋯n:

(1) $$\hat{z}\left(x_{0}\right)=\sum_{i=1}^{n}w_{i}z\left(x_{i}\right).$$

The spatial variable $x_{i}$ represents a vector in a three-dimensional coordinate system. To obtain an unbiased predictor, the weights should sum to one and they are determined by minimizing the variance of the prediction error:

(2) $$w_{i}=\underset{w_{i}}{arg min}\left\{E{\left(\hat{z}\left(x_{0}\right)-z\left(x_{0}\right)\right)}^{2}\right\},$$

where E() denotes the expectation operator. The variance is minimized by the use of the so called semivariogram $\gamma (x_{i},x_{j})$, which is needed to calculate the variance in (2) and can be fitted using the historical dataset of measurements of the quantity $z(x_{i})$ at the sample points. Based on the homogeneity of samples in the area where the random variable $z(x_{i})$ is distributed, its first and second moments are usually assumed to be stationary, which means:

- All random variables have the same mean, that can be estimated by the arithmetic mean of sampled values;
- The correlation between two random variables solely depends on the spatial distance $h=\left|\right|x_{i}-x_{j}\left|\right|$ between them and is independent of their location.

The two above assumptions of stationarity in the random variable are proposed in the geostatistical formalism in order to make the inference of some statistic values possible. Based on the actual measurements of the variables in the different weather stations the required homogeneity of each variable has been heuristically observed.

Under these assumptions the semivariogram is defined as:

(3) $$\begin{array}{c} \gamma \left(x_{i},x_{j}\right)=\gamma \left(h\right)=\frac{1}{2|N(h)|}\sum_{\left(i,j)\in N(h\right)}{\left(z\left(x_{i}\right)-z\left(x_{j}\right)\right)}^{2}, \end{array}$$

where N(h) is the set of pairs of observations *i*, *j*, such that $|x_{i}-x_{j}|=h$, and |N(h)| is the number of pairs in the set. The solution of Equation (2) is obtained using Lagrange multipliers as

(4) $$\left[\begin{array}{c} \hat{W}\\ \mu \end{array}\right]={\left[\begin{array}{cccc} \gamma (x_{1},x_{1}) & \cdots & \gamma (x_{1},x_{n}) & 1\\ \vdots & \ddots & \vdots & \vdots \\ \gamma (x_{n},x_{1}) & \cdots & \gamma (x_{n},x_{n}) & 1\\ 1 & \cdots & 1 & 0 \end{array}\right]}^{-1}\left[\begin{array}{c} \gamma (x_{1},x_{0})\\ \vdots \\ \gamma (x_{n},x_{0})\\ 1 \end{array}\right],$$

where $\hat{W}={\left[w_{1},w_{2},\cdots ,w_{n}\right]}^{T}$ is the vector of estimated weights and μ is the mean value. Commonly used fitting functions of γ(h) are shown in Table 1.

After building the empirical semivariogram, using the available dataset and Equation (3), the next step is to choose the function that will fit it best. As can be seen in table, function expressions contains two unknown parameters $C_{0}$ and $C_{1}$, the distance *h* and a shape parameter θ>0. The shape parameter θ is an hyper-parameter that can be freely selected by the user: different θ values will cause different $C_{0}$ and $C_{1}$ values, which are determined, after selecting θ, with a least square problem resolution.

#### 2.3.2. Radial Basis Function Interpolation

Another commonly used spatial interpolation method relies on the Radial Basis Function (RBF). The predicted variable at an unknown location $x_{0}$ is calculated by the RBF interpolator as:

(5) $$\begin{array}{c} \hat{z}\left(x_{0}\right)=\sum_{i=1}^{n}w_{i}\phi (||x_{0}-x_{i}||), \end{array}$$

where the base RBF function ϕ(h) is defined as:

(6) $$\begin{array}{c} \phi \left(h\right)=e^{-\frac{h^{2}}{\theta^{2}}}. \end{array}$$

After selecting the hyper-parameter θ, the weights $w_{i},i=1\cdots n$ are determined solving the least squares problem:

(7) $$\begin{array}{c} w_{i}=\underset{w_{i}}{arg min}\sum_{k=1}^{n}{\left(\hat{z}\left(x_{k}\right)-z\left(x_{k}\right)\right)}^{2}. \end{array}$$

#### 2.3.3. Natural Neighbour Interpolation

Natural neighbour interpolation is a method developed by Robin Sibson [31], which works with a similar principle to Kriging. The basic equation is:

(8) $$\hat{z}\left(x_{0}\right)=\sum_{i=1}^{n}w_{i}z\left(x_{i}\right).$$

The method differs in the way the weights are calculated, which is based on Voronoi tessellation of the discrete set of spatial points $x_{i},i=1\cdots n$, also called centroids. Voronoi tessellation is a partition of the space into *n* regions (Voronoi cells), where cell *i* consists of all points of the space closer to $x_{i}$ than to any other centroid. We define as $S_{n}\left(x_{i}\right)$ the space region corresponding to cell *i* in the tessellation with *n* centroids, and with $|S_{n}\left(x_{i}\right)|$ the size of cell *i*, which can be the volume or the area of the space region depending on the space being tree-dimensional or two-dimensional, respectively. Given the new location $x_{0}$, a new tessellation is computed using n+1 centroids, adding $x_{0}$ to the previous set of *n* centroids. The Sibson weight $w_{i}$ is calculated as the ratio of the size of the intersection between the new cell $S_{n+1}\left(x_{0}\right)$ and the old cell $S_{n}\left(x_{i}\right)$ with respect to the size of the new cell:

(9) $$w_{i}=\frac{|S_{n}\left(x_{i}\right)\cap S_{n+1}\left(x_{0}\right)|}{|S_{n+1}\left(x_{0}\right)|}$$

### 2.4. Satellite Soil Moisture Modelling

An important step of this project is the SMC modelling. SMC represents the volumetric water concentration and it is expressed as the ratio between the volume of water and and the total volume of considered soil:

(10) $$\mathrm{SMC}=\frac{V_{water}}{V_{soil}}.$$

Typical SMC values goes from 0.0 (completely dry) to 0.6 (fully wet). Values above 0.6 are usually associated with rivers, seas, etc., so they are not related to the soil and have to be discarded. In this project, satellite SMC images are available only twice a week: this depends mainly on the satellite orbit around the Earth. Days without observations have to be “imputed”, which means assigning a reasonable value to the missing observations. To accomplish this goal, an ML model was realized, based on the satellite observations of SMC in the time range of the archived data. The inputs of the SMC model are the output values of the interpolation step, described in the previous section, over the entire Pontecosi area, i.e., the following variables:

- Mean daily temperature on the area;
- Mean daily air humidity on the area;
- Total daily precipitation on the area;
- Mean daily wind speed on the area.

**Figure 7:**
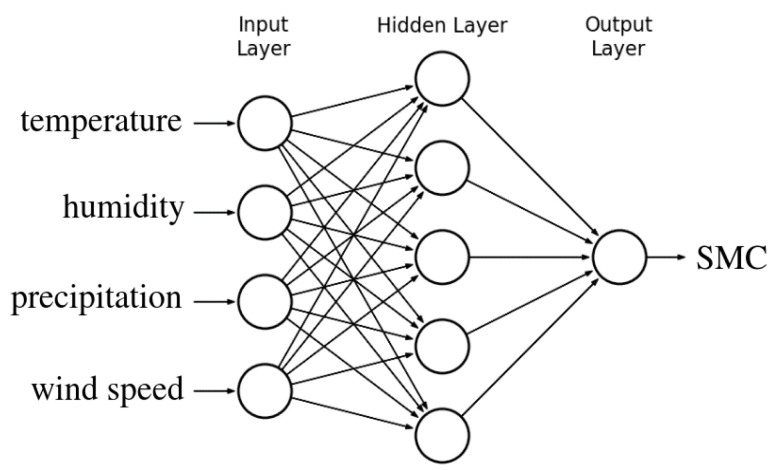
Neural network to predict SMC values.

This was decided thanks to the high availability of this data and the intimate relationship between them and SMC. The target data of the neural network is the average SMC over the basin area obtained from the satellite images. A one hidden layer feed-forward neural network [32] was selected to model SMC as a function of the four weather variables, as shown in Figure 7. The hidden layer uses sigmoidal neurons and the training function employs the Bayesian regularization algorithm [33]. The number of neurons in the hidden layer was chosen through a model selection procedure: a 3-fold cross validation was performed.

### 2.5. Basin Modelling

The core of this work is to predict the hydroelectric basin water level, that strongly depends not only on recent past levels but also on past turbine production and past weather events. According to this, a NARX model was created and tested: the possibility to have as inputs past level values makes the NARX choice suitable to this application.

NARX network is one of most common architectures used to model non-linear processes [34]. The basic principle is that the predicted output of the process $\hat{y}\left(t\right)$ depends on past values both of the input u(t) (that could also be multivariate) and the scalar output itself y(t):

(11) $$\begin{array}{c} \hat{y}\left(t\right)=F(y\left(t-h_{y}\right),y\left(t-h_{y}-1\right),y\left(t-h_{y}-2\right)\cdots ,y\left(t-h_{y}-N_{y}\right),\\ u\left(t-h_{u}\right),u\left(t-h_{u}-1\right),u\left(t-h_{u}-2\right),\cdots ,u\left(t-h_{u}-N_{u}\right)), \end{array}$$

where F() is the non-linear function that describe the process (usually modelled with a neural network), $N_{u}$ and $N_{y}$ are the number of delayed inputs and outputs, respectively, while $h_{u}$ and $h_{y}$ represent the forecast horizons with respect to the input and output, respectively. Equation (11) describes the so called open loop-NARX, while if the predictions $\hat{y}\left(t\right)$ themselves are used in feedback in place of the measured outputs y(t) we have the so called closed-loop NARX:

(12) $$\begin{array}{c} \hat{y}\left(t\right)=F(\hat{y}\left(t-h_{y}\right),\hat{y}\left(t-h_{y}-1\right),\hat{y}\left(t-h_{y}-2\right),\cdots ,\hat{y}\left(t-h_{y}-N_{y}\right),\\ u\left(t-h_{u}\right),u\left(t-h_{u}-1\right),u\left(t-h_{u}-2\right),\cdots ,u\left(t-h_{u}-N_{u}\right)), \end{array}$$

**Figure 8:**
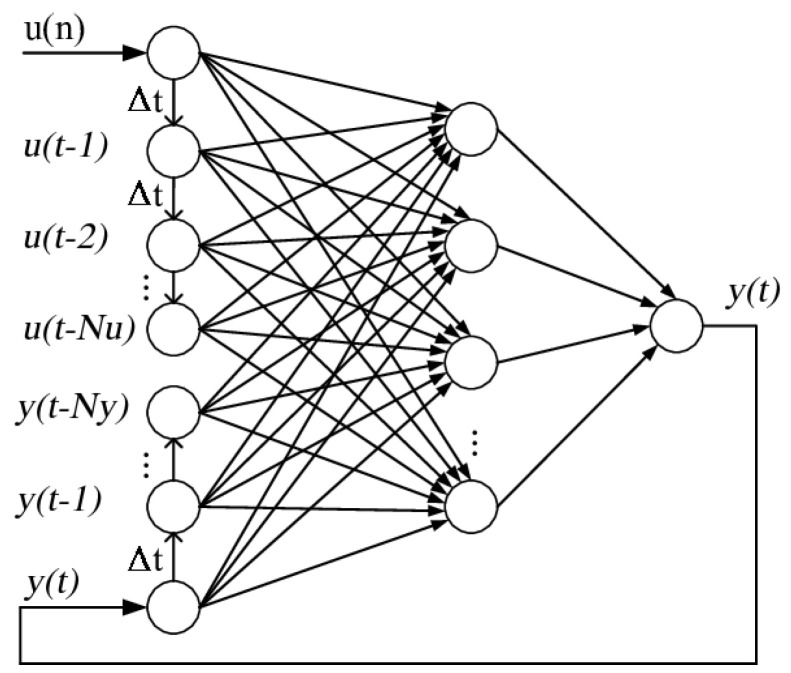
NARX scheme using a neural network.

The neural network is usually trained using the open-loop scheme, while the closed-loop approach can be used at the prediction step to achieve a forecast horizon larger than $\max(h_{u},h_{y})$, as far as the exogenous input u(t) is known. However, the closed-loop scheme can propagate errors worsening the performance. Moreover, training using the closed loop approach may generate instability issues because of the back propagation of the information, while open loop training does not incur into this problem. Figure 8 shows the NARX scheme using a neural network with horizons $h_{u}=0,h_{y}=0$ and a scalar input. The actual model used in this work is better described in Section 3.3, in fact, the vector input contains many variables and particular choices are made for the forecast horizons.

## 3. Results and Discussion

### 3.1. Interpolation of Daily Weather Stations Data Results

The three kinds of interpolators previously described were tested on the weather stations dataset. In particular they were optimized and tested on each of the four daily variables separately.

RBF and Kriging require one hyperparameter, that has to be known in advance, while Natural Neighbour does not require any. The hyperparameter chosen should be the one that generalizes best the spatial distribution of the variable. The procedure used to select hyperparameters is described in the following steps:

1. Hold out one station and consider it as the test station;
2. Perform a leave one out cross validation on the remaining stations and determine the hyperparameter;
3. Test the interpolator on the test station, taking as input all the remaining stations, and calculate the corresponding test error;
4. Repeat the procedure until all the stations had been the test station once;
5. Calculate averaged test error among all test stations.

This procedure was applied for each interpolator and variable, using the mean absolute error (MAE) as loss measure, defined as:

(13) $$MAE=\frac{1}{N}\sum_{i=1}^{N}|{\hat{y}}_{i}-y_{i}|,$$

where N is the number of test observations, $y_{i}$ is the variable and ${\hat{y}}_{i}$ is the predicted value. MAE is a universally accepted forecast error measurement, and it was also the metric of choice of the plant owner. The interpolator with lowest average MAE over all the test stations was chosen as the winning interpolator. This procedure was selected thanks to its robustness: each interpolator has been tested on every station, ensuring that the resulting model is optimized to perform well in each station of the dataset. As shown in Table 2, which reports the results of the above procedure, prediction errors of the Kriging interpolator are small and acceptable. In particular, Kriging, with Gaussian fitting function, was the winning interpolator for each variable. The second best performance is from Natural Neighbour interpolator, while RBF shows the worst performance, especially for the prediction of air humidity and wind speed. A Wilcoxon test for statistical significance of the different results shown in Table 2 was performed, and the test indicated that all differences are significant with 5% confidence. The results of Kriging and Natural Neighbour are very close to each other, while RBF outperforms Natural Neighbour only for the precipitation.

### 3.2. Satellite Soil Moisture Modelling Results

**Figure 9:**
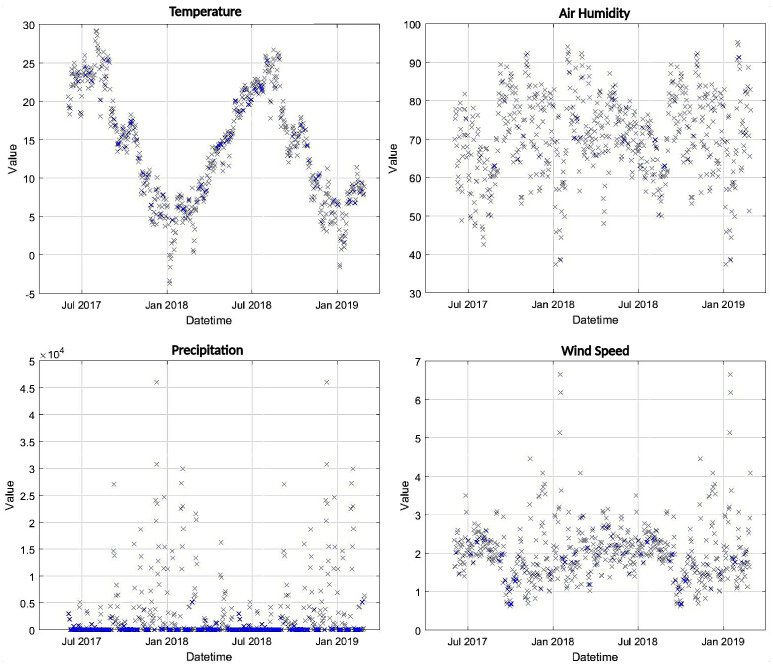
Daily values of the weather variables after averaging the Kriging interpolation over the area of interest.

The Kriging interpolator was used to generate daily values of the four weather variables over a 100×100 grid over the Pontecosi area, as shown in Figure 6. In particular, the interpolated values were generated also for the days when the satellite SMC values are available. All the spatially interpolated values were then averaged to provide a single daily value for each variable, which are shown in Figure 9 over all the dataset time-span. The four variables are then used as inputs to the NN that shall predict the SMC as output.

**Figure 10:**
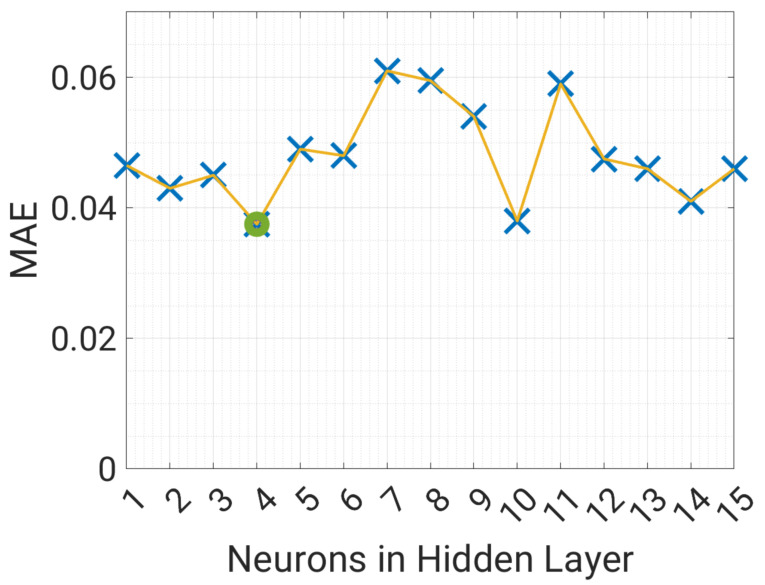
Selection of the number of neurons of the NN to predict SMC.

**Figure 11:**
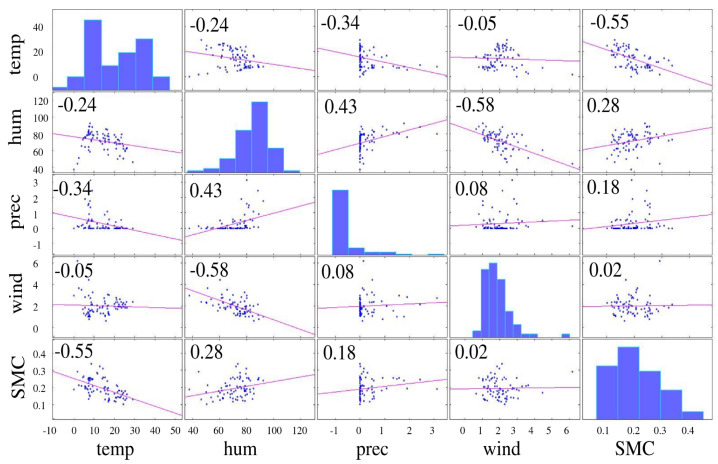
Correlation matrix.

The result of a 3-fold cross-validation is shown in Figure 10. The optimal number of neurons in the hidden layer results to be 4, and the corresponding MAE values are around 0.035. From Figure 10 it is clear that the MAE is nearly independent from the number of neurons, so this choice is not critical, resulting in MAE values between 0.035 and 0.06, depending on the number of neurons. Considering that all observed SMC values are between 0.1 and 0.35 the NN model can be considered accurate. Figure 11 shows the correlation matrix between all the variables, including SMC. In particular, SMC is most correlated with temperature and air humidity, while a low correlation can be observed with the wind speed. On the other hand, the humidity has a strong negative correlation with wind speed (−0.58), as a decrease in air humidity is expected for higher wind speeds, however this has little effect on soil moisture concentration. SMC is expected to be the variable most influencing the water level of the basin, therefore the correlation analysis shows that the other important variables are temperature, air humidity, and precipitation.

### 3.3. Basin Modelling Results

**Figure 12:**
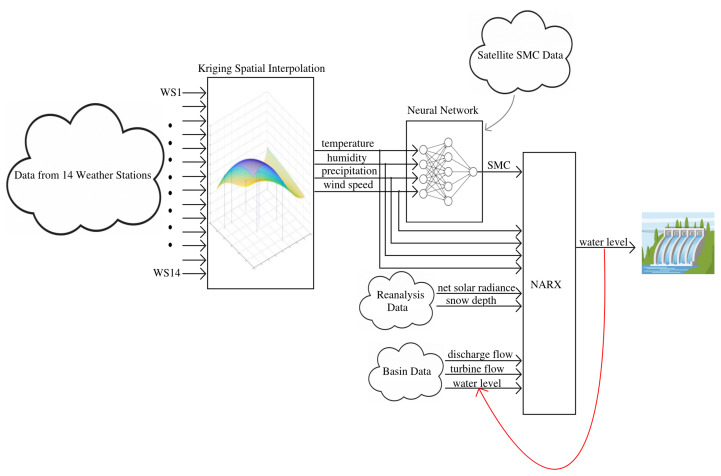
Complete schematic of the forecasting model.

The basin water level was modelled using the NARX model shown in Equation (11). Considering that the basin data (water level, turbine flow rate, and discharge flow rate) are provided hourly, we consider an hourly time-step in the NARX model. The exogenous input $u\left(t\right)\in R^{9}$ is composed by two flow rates and seven environmental variables: four of them are from Kriging interpolation of weather stations (temperature, air humidity, precipitation, and wind speed), two of them are from reanalysis dataset (snow depth, net solar radiance), and the last is SMC as predicted by the trained NN model. All the seven weather variables have a daily temporal resolution, and during day *d* we assume constant intra-day hourly values equal to the last known values of day d−1. Regarding the output water level y(t) it is measured in meters above sea level (a.s.l), and it represents also the tenth input to the NARX model as depicted in the complete scheme of the system shown in Figure 12. The time delays were set to $N_{u}=0$, $N_{y}=0$, while model was trained for different values of the forecast horizon $h=h_{u}=h_{y}=\left\{1,3,6,12,24\right\}$. The resulting NARX equation can be then written as:

(14) $$\hat{y}\left(t\right)=F\left(y(t-h),u(t-h)\right).$$

**Figure 13:**
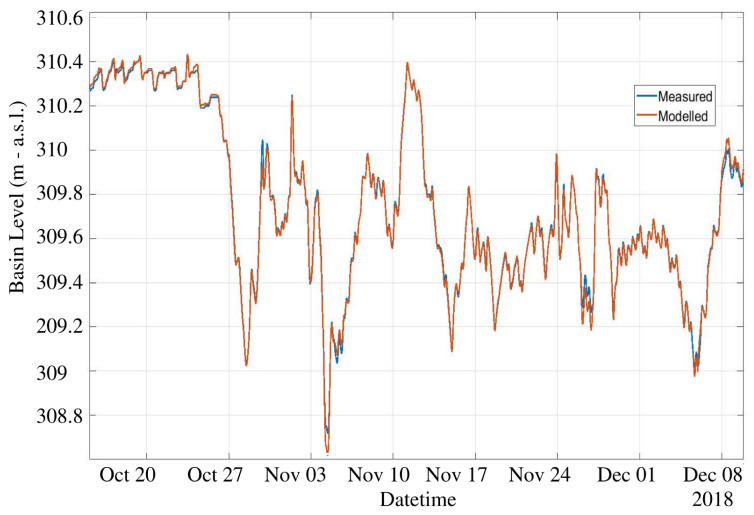
Predicted level during test period, comparison between NARX model and measured for h=1 h ahead. MAE is 2 cm.

**Figure 14:**
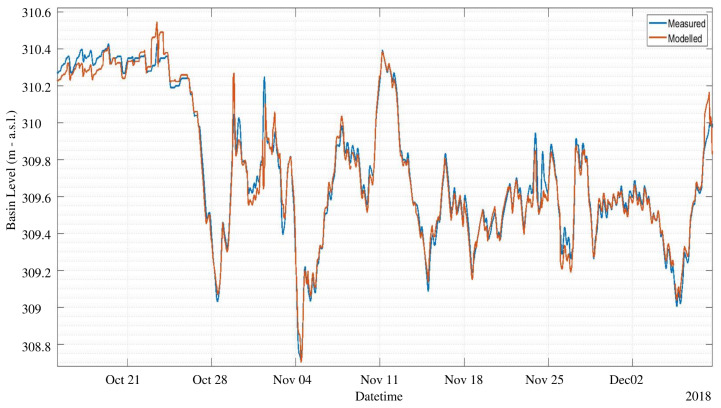
Predicted level during test period, comparison between NARX model and measured for h=6 h ahead. MAE is 10 cm.

**Figure 15:**
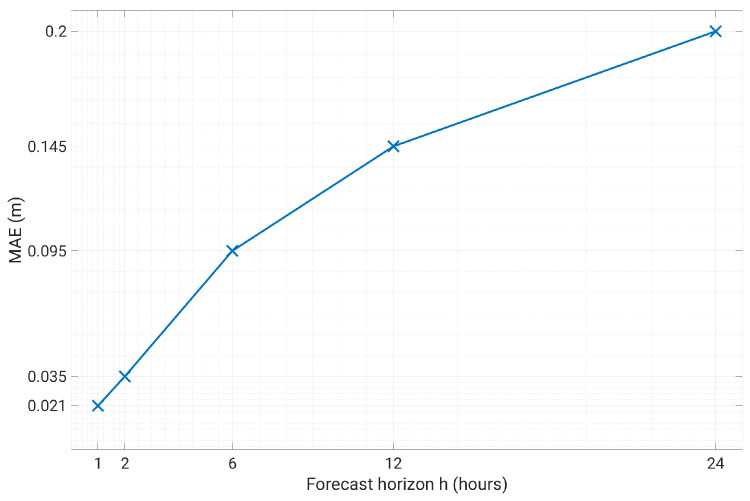
Prediction error as a function of the forecast horizon.

The NARX training period includes the first 500 days of data, and the test set consists in the successive 131 days. We used an expanding window approach: the test predictions during day *d* were obtained including day d−1 in the training set. Figure 13 and Figure 14 show a comparison of the measured level against the predicted level using an horizon h=1 and h=6, respectively. From these figures it can be appreciated that the prediction is able to accurately anticipate even abrupt changes of the water level, such as the one on 27 October, both with 1 or 6 h ahead. The average error is in both cases within few centimeters. Of course, the prediction at 6 h ahead shows a generally worse performance with respect to 1 h ahead, still sudden and significant changes are well predicted. Globally speaking, the mean absolute error MAE in the test set as a function of the forecast horizon is shown in Figure 15. The plant operator assumed as acceptable an error lower than 0.1 m, which is reached with a maximum of 6 h of time horizon. Therefore, our model produces acceptable results for h≤6, while with h=1 the average error is within 0.02 m. If we move to larger time horizons, such as 12 h or 24 h, the average absolute error increases to 15 cm and 20 cm, respectively, indicating a less than linear increase, considering that we have an error of 10 cm with 6 h ahead. For this reason, even the prediction at 24 h ahead can still be considered of interest, as far as an error of 20 cm is small with respect to the decisions that can be made based upon these predictions.

## 4. Conclusions

The objective of this work was to develop a forecasting algorithm to aid a decision support system for the hydrological basin. The complete model is composed of several modules, the last of which is the basin model. To improve the performance, different data sources were used. Weather station data were managed through the use of spatial interpolators: this was necessary because of the large distances between weather stations and basin location. Different types of spatial interpolators were tested and compared, and Kriging was the best one. Soil moisture concentration values in days not available from satellite data were predicted using a neural network. Finally, a NARX model was created to predict the basin water level using past weather and basin data, tacking advantage of the previous modules. Predictions at different time horizons were simulated: acceptable results were achieved within 1 to 6 h ahead. In particular, the basin level can be known up to 6 h ahead with 10 cm accuracy. For larger values of *h*, the model produced larger errors, especially in the case of unpredictable discharge events. If these events are known in advance they can be integrated in the model improving its performance. The methodologies presented in this paper allowed to improve the input data quality and to model accurately an hydroelectric basin. As a future development, the author intend to integrate the model presented in this work with the condition monitoring model previously proposed in [25], also applied to photovoltaic plants [35], in order to improve the performance of the model based on SCADA (Supervisory Control And Data Acquisition) data [36] and self-organizing maps. As a second future development, we shall investigate the application of sensitivity analysis techniques, such as [37,38] to estimate the influence of the input variables to the performance of the model.

## Figures and Tables

**Table 1:** Fitting functions of the semivariogram.

Name	Function
Exponential	$\gamma \left(h\right)=C_{0}+C_{1}\left(1-e^{-\frac{h}{\theta }}\right)$
Gaussian	$\gamma \left(h\right)=C_{0}+C_{1}\left(1-e^{-\frac{h^{2}}{\theta^{2}}}\right)$
Spherical	$\gamma \left(h\right)=C_{0}+C_{1}\left(\frac{3}{2}\left(-\frac{h}{\theta }\right)-\frac{1}{2}{\left(\frac{-h}{\theta }\right)}^{3}\right)$

**Table 2:** Averaged MAE of the interpolation methods.

Interpolator	Temperature	Air Humidity	Precipitation	Wind Speed
Kriging	2.17 °C	8.76%	0.23 mm	1.68 Km/h
RBF	2.81 °C	18.99%	0.25 mm	3.51 Km/h
Natural Neighbour	2.25 °C	11.12%	0.28 mm	2.09 Km/h

## Data Availability

Not applicable.

## Abbreviations

The following abbreviations are used in this manuscript:

NARX	Non-linear Auto Regressive eXogenous input
RBF	Radial Basis Function
EF	Environmental Flow
NWP	Numerical Weather Prediction
ML	Machine Learning
ECMWF	European Centre for Medium-Range Weather Forecasts
IFS	Integrated Forecast System
PWS	Personal Weather Stations
AM	Areonautica Militare
UTM	Universal Transverse Mercator
UPS	Universal Polar Stereographic
SMC	Soil Moisture Concentration
WS	Weather Stations
MAE	Mean Absolute Error
NN	Neural Network
SCADA	Supervisory Control and Data Acquisition
